# Complementary medicine use during cancer treatment and potential herb-drug interactions from a cross-sectional study in an academic centre

**DOI:** 10.1038/s41598-019-41532-3

**Published:** 2019-03-25

**Authors:** Mégane Jermini, Julie Dubois, Pierre-Yves Rodondi, Khalil Zaman, Thierry Buclin, Chantal Csajka, Angela Orcurto, Laura E. Rothuizen

**Affiliations:** 1 Pharmacy Service, Geneva University Hospitals, Geneva, Switzerland; 2 Center for Primary Care and Public Health (Unisanté), University of Lausanne, Lausanne, Switzerland; 3 Institute of Family Medicine, University of Fribourg, Fribourg, Switzerland; 4 Clinical Pharmacology, Department of Laboratories, Lausanne University Hospital, Lausanne, Switzerland; 5 Department of Oncology, Lausanne University Hospital, Lausanne, Switzerland; 6 School of Pharmaceutical Sciences, University of Geneva, University of Lausanne, Geneva, Switzerland

**Keywords:** Cancer epidemiology, Chemotherapy

## Abstract

Complementary medicine (CM) is used by one third to one half of cancer patients throughout the world. The objective of this study was to describe the prevalence of CM use and the potential for interactions with cancer treatments in an academic oncology centre. A cross-sectional study was conducted among patients undergoing current cancer treatment. Among 132 included patients, 56% had used CM since their cancer diagnosis and 45% were using CM during cancer treatment at the time of the survey. The main CM used were green tea (35%), herbal tea (35%), homeopathy (27%), dietary supplements (27%), and herbal medicines (27%). A small majority of patients (58%) spontaneously mentioned the use of CM to their oncologist. Of 42 identified combinations of concomitant use of biologically based CM and anticancer agents among the study patients, the potential for pharmacokinetic interactions of clinical relevance was not expected in 17 combinations (40%), hypothetical and deemed unlikely in 23 (55%), and of probable low clinical relevance in 2 (5%). Considering the high prevalence of CM use, active enquiries should be made by healthcare professionals to detect symptoms that may relate to CM tolerance and effects or that suggest interactions between CM and cancer treatments.

## Introduction

The World Health Organization (WHO) defines complementary medicine (CM) as “a broad set of healthcare practices that are not part of a country’s own tradition or conventional medicine and are not fully integrated into the dominant healthcare system.” In the USA, the 1-year prevalence of CM use was 33.8–42.1% in the nineties^1^. A more recent study showed that in 2007, 33.2% of US adults aged 18 and older had used CM within the past 12 months^2^. In Europe, the prevalence of use in the general population is difficult to estimate because of the heterogeneity of studies and of CM definitions^3^. A recent study in Switzerland showed that 25% of the population aged 15 or older had used CM at least once within the past 12 months^4^.

CM is also widely used among cancer patients throughout the world^5^. Regarding the general population, surveys vary in terms of definitions of CM and types of therapy included in questionnaires, which complicates the assessment of prevalence^6^. In Europe, CM was used by between 15% and 73% of cancer patients^5^. In Switzerland, two studies conducted on oncology patients showed a prevalence of CM use of 26.5% and 39%^7,8^. Swiss mandatory basic health insurance has covered four CM methods since 2012 (traditional Chinese medicine, homeopathy, herbal medicines, and anthroposophic medicine), as long as they are performed by a trained physician. A system of supplemental insurance for expenses not supported by the mandatory basic health insurance also exists, notably covering some CM performed by non-physician therapists.

Users often consider CM as natural, safe, or devoid of harmful potential^6,9,10^. Yet some risks linked with the use of biologically based CM^11^ (e.g. herbal products and other natural products) do exist, potentially translating into adverse events and drug interactions^6,9^. Biologically based CM-related adverse events are not well documented, and available data mainly depend on spontaneous notifications by users^12,13^. Given that up to 70% of patients tend to not spontaneously disclose their CM use to their oncologist^14^, there is a risk that CM-related adverse events will not be detected or will be confounded with side effects related to cancer treatments. These adverse events can be of variable degrees of seriousness^6,13^. Most frequently, allergic reactions or gastrointestinal problems may occur^5^, but occasional serious adverse events have been reported in the literature, including cases of hepatotoxicity, neurotoxicity, or nephrotoxicity^6,15–17^. Interactions can lead to different possible consequences. Toxicity can result from pharmacokinetic potentiation or addition of pharmacodynamic effects, but decreased efficacy is also possible if a pharmacokinetic interaction results in decreased exposure to a cancer drug concentration or if the CM interferes at the anticancer agent effector site^6^.

The best known metabolic or transport pathways associated with pharmacokinetic interactions of significant clinical relevance are hepatic P450 cytochrome isoenzymes, glucuronidation enzymes^18–20^, and transmembrane transporters at the biliary, renal, or intestinal levels (PgP, MRP1–2, AOT, OCT)^21^. Thus, cancer treatments that seem to hold the highest risks of interaction are conventional chemotherapies and certain targeted therapies (e.g. tyrosine-kinase inhibitors) whose disposition depends on those pathways. In addition, concerns have been raised that hormonal properties attributed to some CM may influence the efficacy of anticancer hormonal therapies or promote the progression of hormone-dependent cancers themselves^19,22^. Consultant requests in clinical pharmacology regarding the potential for interactions between biologically based CM and drug therapies are frequent in our academic hospital, especially in areas such as oncology, transplantation, and infectious diseases.

The objectives of this study were twofold: first, to describe the prevalence and characteristics of CM use in patients currently undergoing cancer therapy in the outpatient setting of a Swiss academic hospital oncology centre, as well as to explore communication about CM use between patients and physicians or nurses; and second, to evaluate the risk of interactions between cancer treatments and the CM used by these patients from a thorough search of the available literature.

## Results

**Figure 1:**
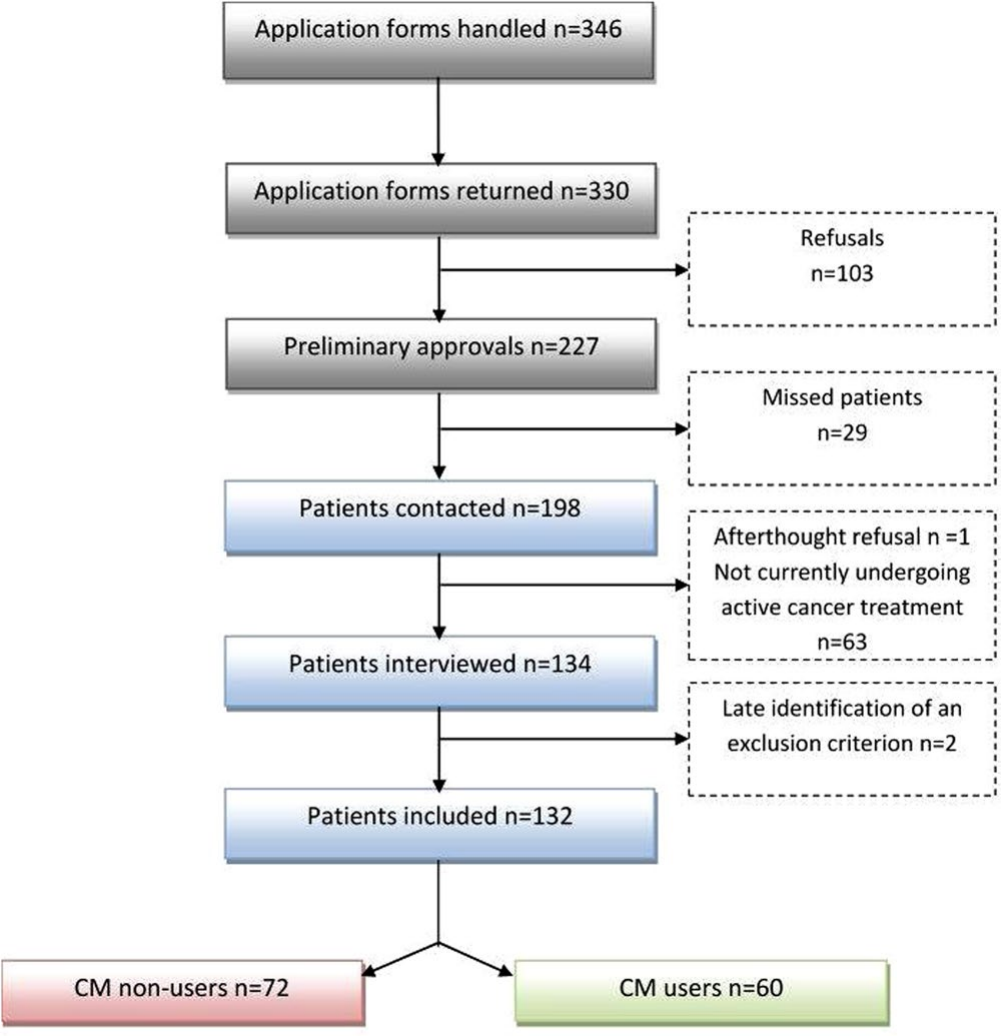
Patients’ recruitment procedure. CM = complementary medicine.

A total of 134 patients answered the questionnaire but, because of the late discovery of exclusion criteria in the medical files, 132 patients were included in the final analysis. As the study was investigator-dependent in terms of availability for carrying out the interviews at the end of oncology visits, some patients who had given preliminary approval were missed for the interview. Details of the enrolment steps are given in Fig. 1. Socio-demographic characteristics and medical data are described in Tables 1 and 2, respectively.

**Table 1:** Patients’ socio-demographic characteristics.

	Total (N = 132)	CM users N = 60 (45%)	CM non-users N = 72 (55%)	P-value
**Age (years)**				0.007
Median (range)	62 (86–20)	59 (79–20)	65 (86–34)	
Mean (SD)	62 (13)	58 (14)	65 (11)	
**Sex**				0.22
Male	47 (36)	18 (30)	29 (40)	
Female	85 (64)	42 (70)	43 (60)	
**Origin**				0.36
Europe	125 (95)	55 (92)	70 (97)	
Africa	4 (3)	3 (5)	1 (1)	
USA	3 (2)	2 (3)	1 (1)	
**Educational level**				0.06
Basic	8 (6)	2 (3)	6 (8)	
Apprenticeship	69 (52)	28 (47)	41 (57)	
High school/college	7 (5)	4 (7)	3 (4)	
University	48 (37)	26 (43)	22 (31)	
**Marital status**				2.63
Single	24 (18)	12 (20)	12 (17)	
Married/widowed/civil partnership	86 (65)	41 (68)	45 (62)	
Divorced/separated	21 (16)	6 (10)	15 (21)	
Unknown	1 (1)	1 (2)	0 (0)	
**CM health insurance**				0.001
Covering CM	48 (36)	32 (53)	16 (22)	
Not covering CM	67 (51)	24 (40)	43 (60)	
Do not know	17 (13)	4 (7)	13 (18)	

CM: complementary medicine.

Results are expressed as number of participants (percentage) unless otherwise indicated.

**Table 2:** Patients’ cancer type, stage, and current treatment.

	Total (N = 132)	CM users N = 60 (45%)	CM non-users N = 72 (55%)	P-Value
**Median weight in kg (range)**	70 (117.5–43.5)	76.3 (117.5–43.5)	72 (114.5–45)	0.59
**Cancer type**				0.53
Breast	41 (31.1)	19 (31.7)	22 (30.6)	
Lungs	14 (10.6)	7 (11.6)	7 (9.7)	
Gastrointestinal	14 (10.6)	4 (6.7)	10 (13.9)	
Pancreas	9 (6.8)	7 (11.6)	2 (2.8)	
Brain	8 (6.1)	6 (10.0)	2 (2.8)	
Lymphoma	8 (6.1)	4 (6.7)	4 (5.6)	
Ovaries	6 (4.5)	1 (1.7)	5 (6.9)	
Prostate	6 (4.5)	1 (1.7)	5 (6.9)	
Skin	4 (3.0)	1 (1.7)	3 (4.1)	
Others	22 (16.7)	10 (16.6)	12 (16.7)	
**Stage** (**TNM)**				
Local advanced (T3-T4)	67 (51)	32 (54%)	35 (49%)	0.57
Nodal metastasis	67 (51)	30 (50%)	37 (51%)	0.98
Distant metastasis	48 (36)	20 (33%)	26 (36%)	0.93
**Current anticancer treatment** ^**a**^				
Chemotherapy	82 (62)	38 (63)	44 (61)	0.79
Targeted therapy	42 (32)	22 (37)	20 (28)	0.27
Hormonal therapy	30 (23)	12 (20)	12 (20)	0.49
Radiotherapy	9 (7)	2 (3)	7 (10)	0.22

CM: complementary medicine.

Results are expressed as number of participants (percentage) unless otherwise indicated.

^a^Numbers total more than 100% because some patients underwent several therapies at the same time.

CM users were significantly younger than non-users (median age 59 versus 65 years, respectively; P = 0.007). Supplemental insurance coverage for CM was significantly more frequent among CM users (57%) than among non-users (27%, P = 0.001), but 13% of participants did not know whether they had subscribed to such healthcare coverage or not. No significant correlations were found between use and non-use of CM regarding the distribution of cancer types (organ), the seriousness or extent of cancer (localized or metastatic), or the type of cancer treatment. A total of 45 different anticancer agents were administered in the studied population. The most frequent cancers were breast, lung, and gastrointestinal (31%, 11%, and 11%, respectively). The lifelong prevalence of CM use by participants was 75% (n = 99), since cancer diagnosis was 56% (n = 74), and while undergoing cancer treatment at the time of the survey was 45% (n = 60). The prevalence of biologically based CM use during treatment was 36%, which represents 78% of all CM users. CM users reported 29 CM types. A majority of patients (76%) used several CM types, and some used several CM of the same type (e.g. more than one homeopathic preparation or more than one herbal medicine). Overall, the total number of uses of CM types by our participants was 175. When we took into account each CM use of the same type (e.g. two homeopathic preparations taken by the same patient), the number of CM uses rose to 199. A majority of patients used green tea, herbal teas, homeopathy, dietary supplements (vitamins, minerals, and oligo elements), herbal medicines, and traditional healers (Table 3).

**Table 3:** Types of complementary medicine.

Complementary medicine	Current users (N = 60)
**Biologically based**	
Green tea*	21 (35)
Herbal tea**	21 (35)
Dietary supplements	16 (27)
Herbal medicine***	16 (27)
Aromatherapy	8 (13)
Omega 3	1 (2)
**Non-biologically based**	
Homeopathy	16 (27)
Traditional healer	11 (18)
Acupuncture	6 (10)
Lymphatic drainage	5 (8)
Naturopathy	4 (7)
Tai chi	4 (7)
Osteopathy	4 (7)
Therapeutic massage	4 (7)
Reflexology	3 (5)
Hypnosis	3 (5)
Meditation	3 (5)
Yoga	3 (5)
Shiatsu	3 (5)
Others	23 (38)
Total	175

Results are expressed as number of participants (percentage). The total represents the number of uses of complementary medicine (CM) types combined. Only biologically based CM purchased for medical purposes were included.

*Green tea: tea made from the cured leaves of the tea tree (*Camellia sinensis*).

**Herbal tea: infusions, decoctions, or macerations of leaves, flowers, fruits, stems, seeds, or roots from plants other than *Camellia sinensis*.

***Herbal medicine: any herbal CM other than tea or herbal teas (e.g. dry extracts as pills or capsules, tinctures, *Viscum album* injections, etc.).

### Motivations for use and perceptions related to CM

In approximately one third of cases, the CM was used with the intent of reducing adverse effects linked to cancer treatments (32%). Reinforcement of the body by improving its immunity (21%) and treating cancer (15%) were also important motivations for the use of CM. Regarding biologically based CM, green tea and herbal tea were mainly used for hydration (63% and 52% of users, respectively), dietary supplements were used to treat cancer (39%) and to improve immunity (19%), and herbal medicines were used to reduce adverse events (41%) and to improve immunity (26%).

**Figure 2:**
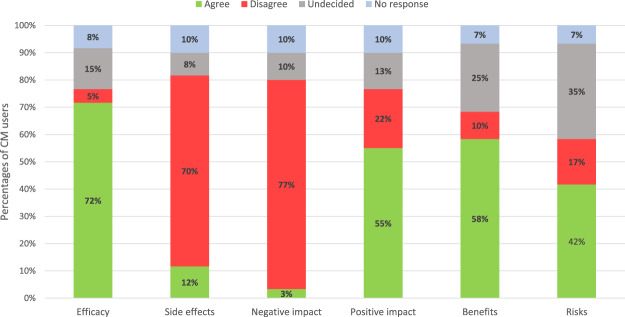
Patients’ perception of complementary medicine (CM). Results are expressed as the percentage of CM users agreeing more or less with questions that explore the belief in the efficacy of CM, their potential side effects, and their positive or negative impacts on cancer or its treatment. The last two items depict the extent to which patients deem themselves sufficiently informed about the benefits and risks associated with CM.

A majority of users (72%) considered their CM therapies effective, and most CM users (70%) did not think that CM could induce any side effects. They also did not think that CM could have a negative impact on their current cancer treatment (77%). Over half of users (55%) thought that their oncological treatment could be positively reinforced by CM, either directly or indirectly. In addition, they considered themselves sufficiently informed about the benefits expected from the CM they used (58%). Forty-two percent considered themselves sufficiently informed about the risks related to CM use, whereas approximately one third remained undecided on that point (35%) (Fig. 2).

### Reasons for not using CM

Among CM non-users, a majority (56%) claimed that they had always been in good health before cancer and that they had trust in conventional medicine and in their physician. Some also feared that CM might interfere with their treatment or their cancer (13%), did not believe in the efficacy of these therapies (24%), were not interested in them (14%), or did not know about CM (11%). Six patients mentioned financial-based reasons for not using CM (8%) and most did not have supplemental insurance with CM coverage.

### Communication about CM

**Figure 3:**
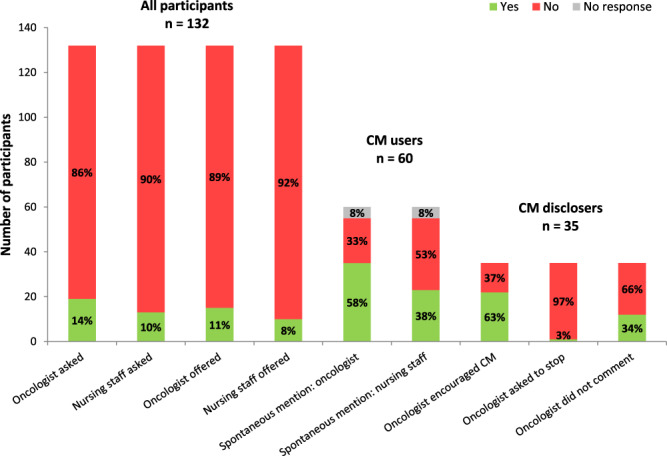
Communication about complementary medicine (CM). The first two bars indicate the percentage of participants asked by either the oncologists or the nursing staff if they were currently using CM. The third and fourth bars indicate the percentage of participants to whom oncologists or the nursing staff offered CM as part of care. The next two bars indicate the percentage of CM users who spontaneously mentioned their CM use to their oncologists or to the nursing staff. The last three bars indicate the reaction of oncologists to the disclosure of CM use by participants.

When asked about communication with the medical staff about CM use, 113 patients (86%) declared that their oncologist did not ask whether they were currently using CM or not. Most (n = 117, 89%) also answered that their oncologist did not discuss offering CM as part of care. Trends were similar regarding communication with the nursing staff, with no enquiry as to current CM use reported by 119 patients (90%) and no CM offered as part of their care according to most patients (n = 122, 92%). Thirty-five CM users (58%) spontaneously mentioned their use of CM to their oncologists, and 23 mentioned it to the nursing staff (38%). The oncologists who were informed about their patients’ CM encouraged patients to go on with it in 63% of cases, whereas they did not offer any recommendation in 34% of cases. Only one patient stated that his oncologist asked him to stop using CM, which consisted of green tea and borage extract (Fig. 3). Half of the CM users who did not mention their use to their oncologist (n = 10) considered this information not important and felt that it did not concern their oncologist.

### Interactions between CM and cancer treatment

**Figure 4:**
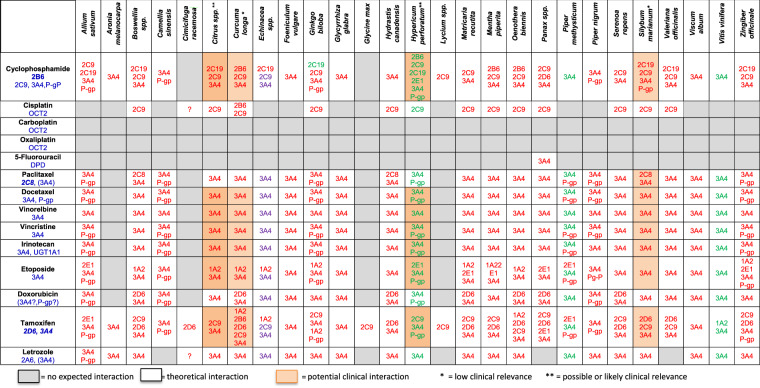
Table of potential pharmacokinetic interactions between herbal medicines and anticancer agents. Properties of herbal medicines in metabolic/transport pathways: Red = inhibition, green = induction, violet = controversial in references (inhibition and/or induction), blue: metabolic/transporter pathway, italic blue*** = ***metabolic pathway leading to a major active metabolite; 3A4 = cytochrome P450 3A4 (etc.); P-gp = P-glycoprotein; OCT = organic cation transport; DPD = dihydropyrimidine dehydrogenase. References used to build the table can be found online in Supplementary Data S3 and Supplementary Table S4.

Seventy-five articles were selected in order to assess the interaction potential of biologically based CM seen in our survey and to develop a table. Forty-four articles discussed CM that were recurrently cited as presenting documented or theoretical potential for interaction. The other 31 articles more specifically explored the biologically based CM encountered in our patients and were used to assess potential two-by-two CM-anticancer agent interactions (see Appendix 1). Nineteen herbal products were regularly cited in the literature as presenting potential risks of interaction, mostly through interference with liver enzymes or transporters, and sometimes through unknown or non-pharmacokinetic mechanisms (e.g. antioxidant properties or pharmacodynamic actions such as platelet aggregation inhibition). However, among these herbal products, only four were documented as perpetrators of pharmacokinetic interactions with a significant risk of clinical impact (*Citrus* spp., namely grapefruit; *Hypericum perforatum* or St. John’s wort; *Silybum marianum* or milk thistle; and *Curcuma longa* or turmeric). The comprehensive search for interaction potential was extended to 12 biologically based CM used by our patients that did not appear in the literature as CM classically associated with a risk for interaction. Among these, there were nine herbal CM as follows: *Boswellia* spp. (incense tree), *Linum usitatissimum* (flaxseed), *Aronia melanocarpa* (Aronia berry), *Lycium* spp. (goji berry), *Viscum album* (mistletoe), *Zingiber officinale* (ginger), *Matricaria recutita, Foeniculum vulgare* (fennel), and *Mentha piperita* (peppermint). No interactions of clinical relevance were retained for these substances. It was not possible to distinguish between a lack of data due to the absence of available studies or an absence of interaction potential as shown by appropriate studies. Thus, their interaction potential remains theoretical at best, based on hypothetical mechanisms or *in vitro* or animal data. Non-herbal biologically based CM used by our patients (vitamin C; melatonin; royal jelly; *Arthrospira* spp., namely, spirulina; and glucosamine) were also overviewed, but they are not detailed further, as no significant pharmacokinetic interactions were expected. Finally, *Linum usitatissimum* (linseed or flaxseed), as well as the anticancer agents gemcitabine, anastrozole, methotrexate, and monoclonal antibody therapies, were omitted from the table for simplification purposes, as no pharmacokinetic interactions were expected for the studied combinations. Overall, only herbal CM were identified as potential perpetrators or victims of pharmacokinetic interactions in our study. Our interaction table (Fig. 4) includes 378 theoretically possible encounters between 27 biologically based herbal CM and 14 anticancer agents. Among these encounters, we found four different categories of interactions: no expected interaction (128 pairs), only a theoretical interaction risk (unlikely clinical relevance, 221 pairs), a seemingly low clinical interaction relevance (15 pairs), and a possible or likely clinical interaction relevance (14 pairs).

In the table, we identified 42 combinations of concomitant use of an herbal CM and an anticancer agent by our patients. Among these associations, 17 (40%) revealed no interaction risk, 23 (55%) only a hypothetical interaction risk, 2 (5%) a potential clinical interaction of seemingly low relevance, and none an interaction risk that was deemed clearly problematic. Of the two potential clinical interactions, one in particular was of concern from a theoretical point of view: the concomitant use of *Hypericum perforatum* (St. John’s wort) and letrozole. St. John’s wort is a powerful cytochrome P450 3A4 inducer and this pathway is cited as one of letrozole’s metabolic routes. This suggests that the combination could lower letrozole concentration exposure with an impact on cancer treatment efficacy^20,23,24^. However, no significant pharmacokinetic interactions were reported to date with other strong CYP 3A4 inducers or inhibitors, or observed when cimetidine, a CYP 3A4 inhibitor, was co-administered with letrozole, suggesting that CYP 3A4 may be only a minor pathway of letrozole metabolism among other metabolic routes (e.g. CYP 2D6). Therefore, this interaction, which was initially considered possibly clinically relevant, was later downscaled to only theoretical. The other potential clinical interaction involved paclitaxel and *Curcuma* extracts, known to inhibit certain CYP isoforms and to increase paclitaxel exposure at high doses in animal experiments, but with uncertain translation in clinical conditions.

## Discussion

The prevalence of CM use among our study patients was 45% during cancer treatment. The most used CM were biologically based. Compared with non-users, CM users tended to more often be female and younger and to benefit from health insurance coverage for CM. Patients who mentioned using CM declared a willingness to play an active role in their care. Only two interactions of probable low clinical relevance between biologically based CM and anticancer agents were found among all study patients.

The prevalence of CM use among oncology patients during this study was higher than in studies conducted in a neighbouring academic hospital (26.5%)^8^ and in a region of the German-speaking part of Switzerland (39%)^7^. However, it was within the range of 33–60% that was found in other European countries between 2011 and 2014^19,25–28^. Our results showed that patients mostly used herbal teas, green tea, herbal medicines, dietary supplements, and homeopathy. Similar results were reported in a Swiss German study regarding the use of homeopathic treatment^7^ and in a German study regarding the use of dietary supplements, homeopathy, and acupuncture^26^. The use of dietary supplements was similar in an Italian study, whereas homeopathy, herbal medicines, and acupuncture were more frequently encountered among our patients^25^. The use of herbal medicines was lower in our study compared with that in a Costa Rican study, where they were taken by half of oncology patients during their cancer treatment^29^. In the United Kingdom and the USA, vitamins and minerals were frequently used^19,30,31^. For example, Patterson, *et al*.^30^ found that 63.5% of the oncology patients included in their study had used vitamins/minerals in the past year, whereas in our study, only 27% used such substances. In Germany and in the German-speaking part of Switzerland, mistletoe was often used to strengthen body defences and to fight cancer, probably related to a prevalent anthroposophic tradition^5,27^. In our study, only two patients used mistletoe, in line with the limited representation of anthroposophic medicine in the French part of Switzerland. Regarding green tea and herbal teas, many patients used them on a daily or regular basis, but their motivation was rarely for assumed curative properties; most considered them instead as part of their usual diet. When not taken for a medical purpose, these teas were not recorded as CM in our study. We did not find any explicit information on the prevalence of herbal tea and green tea use in the literature. It is likely that most studies excluded them, as it is hard to assess whether they are really considered as CM by patients. Products from the tea tree, specifically green tea, are rich in polyphenols and are widely believed to have cancer preventive effects in relation to their antioxidant properties^32,33^. Green tea and herbal teas for medical purposes were both taken by one third of our CM users. Therefore, we decided to include them in our study, thereby allowing for their inclusion in our interaction risk evaluations with anticancer agents^34^.

CM users in our population tended to be younger than non-users, in line with other studies conducted in Switzerland and Europe^5,7,8,25,27^. Besides a younger age, we observed a trend, although not statistically significant, in favour of women and of individuals with a higher education level among CM users. Other studies have shown that female gender, younger age, and higher educational background were predictive of CM use during cancer treatment^14,25,31^. We hypothesize that patients with a higher education level are better informed about existing care options and have more financial resources that allow them to afford CM treatments^31^. Our results showed a statistically significant difference between CM users and non-users regarding health insurance coverage for CM, with CM users’ insurance more often covering CM expenses. Causality is probably bidirectional here: patients likely to use CM might more easily subscribe to a specific health coverage for it, and patients whose CM expenses are covered probably use these therapies more frequently.

CM users seemed satisfied with CM, as they considered that they met their needs and were effective. Most patients considered CM to be harmless and safe and felt that these therapies could potentiate the efficacy of anticancer agents and reduce their adverse effects. Paul^27^ and Molassiotis^5^ reported that one of the major goals of patients using CM was to increase the ability of the body to fight cancer by strengthening the immune system. In our study, this reason came second behind the goal to reduce adverse effects and symptoms caused by cancer treatments, as also noted in a survey conducted by Tascilar^6^. Those who use CM may show a willingness to contribute to their own care and to control the disease, as observed in other studies^5–7,26,30,35,36^.

A slight majority of patients spontaneously mentioned their CM use to their oncologist, which was somewhat higher than in other studies in which half of the patients did so^5,37^; still, our results are consistent with those of an Italian study^25^. Disclosure to nursing staff was lower than it was to oncologists. Most patients who disclosed their CM use were encouraged by their oncologist to go on with it. Oncologists are in a good position to identify patients who need advice on CM or are at risk in the context of oncology treatment and concomitant CM use. Oncologists should thus redirect patients to qualified interlocutors within the hospital, for example, integrative physicians, who could give them specific advice on the benefits and risks of CM. Open-mindedness, an understanding attitude, and, above all, communication with the patient are key elements to identify and prevent misinformation, potential adverse effects, and interaction risks^38–40^. In this study, patients reported that most oncologists and nurses of the ambulatory oncology centre did not ask them about CM use and rarely mentioned CM therapies. Because the low rate of questions by the medical staff could be linked with their lack of knowledge about CM^41^, better education programs should be developed for them. As suggested by Ben-Arye, *et al*.^42^, “all herbal medicinal products being used should be recorded in the medical file, and patients should be referred to a qualified IP [Integrative physician, trained in herbal medicine] for guidance. The consultation should be part of the conventional oncology service, and patients should be asked specifically about their use of herbal medicine, as well as about their expectations from this treatment.”

Compared with a recent Costa Rican study^29^, in our study we found fewer theoretical interactions, which could be linked with a lower rate of herbal medicine use in our sample. However, herb-drug interactions with potential clinical risk were similar in both studies. Scientific data on potential interactions between CM and cancer treatments are scarce, most of the time limited and vague, and sometimes contradictory. The lack of standardized content of herbal products, the possible presence of numerous components, and the diversity in regimens contribute to the difficulty in studying and quantifying interaction risks with herbal treatments. It is thus difficult to answer clinicians’ practical questions with reliable and relevant information. From a clinical point of view, the most acknowledged problematic association is the concomitant use of St. John’s wort (*Hypericum perforatum*) or grapefruit (*Citrus* spp.), as they are potent inducers and inhibitors of CYP, respectively, and anticancer agents are metabolized through the CYP 3A4 pathways. Concomitant use leads to a significant risk of reduced efficacy or increased toxicity of these anticancer agents^43,44^. Out of prudence, even in cases where the interaction is deemed only theoretical, we do not recommend that oncology patients take St. John’s wort or grapefruit concomitantly with their cancer treatment. Although the risk is deemed low, the use of milk thistle extracts (*Silybum marianum*) with CYP2C8, 2C9, 2C19, 2D6 and 3A4 substrates is best avoided as a measure of caution, as studies remain insufficiently conclusive about whether there might be significant CYP inhibition on a clinical level with the use of this CM^45–47^. The true clinical relevance of interactions with curcuma (*Curcuma longa*) is also yet to be determined. Neither grapefruit nor milk thistle was reported by our CM users concomitant with their cancer treatment.

Field experience from our clinical pharmacology unit suggests that most patients use herbal products that are absent from the lists of “classic” substances having interaction risks. Indeed, herbal products with a potential interaction described in the literature were not those used by our study patients. This might result from the patients being warned by their physician, especially their oncologist, of the potential risk linked to these herbal extracts if taken with their cancer treatment. On the other hand, biologically based CM are characterized by the regular renewal of trends and products -often regulated as dietary supplements and not as drugs-, with the consequent risk that clinically relevant interactions with cancer therapies might not yet be known. This emphasizes the importance of pharmacovigilance, a public health activity that suitably complements specialized pharmacological consultation.

This study has several limitations. First, our broad definition of CM, excluding only supermarket products purchased for no medical purpose, and the limited size of the sample might have had an influence on the results and could explain some differences from other studies. Second, our study focused on an outpatient service of an academic hospital located in an urban area. It is thus possible that the studied population did not completely reflect the adult oncology population living in more rural regions or being treated in non-academic oncology centres. Third, certain types of cancer treatments (e.g. tyrosine kinase inhibitors) were not represented among CM users in our study and were therefore not included in our discussion, although they probably represent a source of concern with respect to interaction risks with herbal CM.

In conclusion, 45% of the patients treated for cancer used CM alongside cancer treatments. Thus, the role of the oncologist and other physicians is essential in understanding patients’ reasons for CM use in order to prevent interactions and identify adverse effects. Regarding pharmacokinetic interaction risks, no associations between CM and cancer treatments in our study population were found to be clinically worrisome. However, in recent years, no new CM has been added to the very short list of CM herbal products acknowledged to be linked with clinically relevant interactions (e.g. St. John’s wort, grapefruit). Promoting an active pharmacovigilance strategy for CM could help generate better evidence, for example, by documenting both the presence and absence of problems during CM use and by encouraging patients to contribute by reporting CM use and adverse events. Dispensing specific education and tools to healthcare providers about which CM could help patients, given their individual conditions and symptoms, as well as about the interaction potential of CM, would be useful, but such tools hold practical limitations (e.g. the vast number of substances and the rapid progress in oncology treatments). Development of integrative medicine centres in oncology services could promote interdisciplinary collaboration between patients, physicians, oncologists, and researchers specialized in this field.

## Methods

### Design and setting

This observational cross-sectional comparative study was conducted at the ambulatory oncology centre of the Lausanne University Hospital in Switzerland over a 6-week period from March to April 2014. At the time of the study, no CM was available to patients as part of integrative oncology at this oncology centre. Data were collected through structured interviews that followed a 27-item questionnaire, with subsequent examination of the patients’ medical files. The study was approved by the Cantonal Commission for the Ethics of Human Research (CER-VD) (reference number: 41/14). All research was performed in accordance with relevant regulations.

### Participants

Any adult patient (>18 years old) who agreed to be interviewed and was currently undergoing cancer treatment, i.e., administered between 10 days preceding and 10 days following the interview, was eligible for the study. Intermittent therapies (e.g. chemotherapy, radiotherapy) and long-term or maintenance therapies (e.g. imatinib, hormonal therapy) qualified as cancer treatment for this study. Because of the organizational aspects of the study, most patients from specialized consultations in haemato-oncology, dermato-oncology, or sarcoma were not included, but a few patients with lymphoma or melanoma were followed in the general oncology outpatient clinic and could thus be included in the study. Exclusion criteria were cognitive impairment, altered ability to consent, or any situation compromising communication or comprehension of the project (e.g. language barriers). All consecutive patients consulting the ambulatory oncology centre received written information about the study, along with a form asking whether they agreed to be informed about it. An investigator met those who gave their preliminary agreement and explained the study. Patients who then agreed to take part signed the informed consent and were interviewed at the end of their oncology visit.

### Variables

In the absence of a validated questionnaire, the questionnaire was developed by a team of oncologists, pharmacists, and family physicians on the basis of other published questionnaires^14,48^. It was composed of 27 questions, mostly closed ended. The definition of CM used in this study was based on the WHO definition. Because this definition of CM is broad, we excluded supermarket products purchased for no medical purpose in order not to falsely increase the prevalence of herbal tea use. CM categories were taken from the initial National Center for Complementary and Alternative Medicine structural classification^11^ still in use today^49^, with biologically based CM considered separately from non-biologically based CM categories (alternative medical systems, mind-body interventions, manipulative and body-based methods, and energy therapies). Biologically based CM include herbal or other natural products such as minerals, hormones, and biologicals. Tea made from the leaves of the tea tree (*Camellia sinensis*) was distinguished from herbal teas, defined as infusions, decoctions, or macerations from plants other than *Camellia sinensis*. Herbal medicines included any herbal CM other than tea or herbal teas, such as dry plant extracts taken as pills or capsules, tinctures, or injections (*Viscum album*). However, CM types resulting from extreme dilutions (such as homeopathic therapy, spagyrics, or Bach flower remedies), which are not expected to contain pharmacologically relevant amounts of the products from which they originate, are included among non-biologically based CM. Classification of aromatherapy appears to be not clearly defined. Because it originates from natural products and is used with the intent of it having a central chemical effect through the stimulation of olfaction, aromatherapy was included in the biologically based CM.

The first set of questions investigated socio-demographic and insurance-related aspects. The second set of questions explored characteristics of CM use, such as type of CM, sources of information on CM, and patients’ perception of risks and benefits of CM. The third set of questions investigated communication about CM use shared by the patient with the medical team. The medical files of each patient who answered the questionnaire were screened in order to collect information on cancer diagnosis (type, stage) and cancer treatments (past and ongoing treatments, including specific substances, dosages, and dates of cancer treatment administration).

### Interaction table

First, a literature search on existing data about CM that are known, or suspected, to interact with anticancer agents was performed. Because system-wide exposure is expected from biologically based CM delivered through gastrointestinal (enteral) or parenteral routes (e.g. intravenous or intramuscular), these CM present a risk of interactions with ongoing enteral or parenteral cancer therapies. Any relevant interaction risk detected during interviews or from the medical files was to be reported to the oncologist, provided the patient had previously given consent for this reporting. Second, biologically based CM that were identified through the interviews as being used by our patients but that were not known, or suspected, to have an interaction potential were also explored for this by using the same search method. To build the interaction table, we reviewed in detail articles on the interaction potential of the CM defined above and cancer treatments taken by the CM users included in this study. The pharmacokinetic characteristics (metabolic and elimination pathways) of anticancer agents were taken from two pharmacology reference books^50,51^ and the medicinal product information (drug monograph) approved by the Swiss agency for therapeutic products (Swissmedic). The literature search was performed with MEDLINE by using the following keywords: [anticancer agent’s name] and “drug interaction”; [anticancer agent’s name] and CYP450 interaction; [anticancer agent’s name] and pharmacokinetics; [anticancer agent’s name] and pharmacodynamics; complementary medicine and drug interaction: [CM substance name] and drug interaction; herb drug interaction.

An interaction was considered not expected when no common metabolic pathway or transport route, nor published data suggesting an interaction, was identified for the CM and the cancer drug combination. The interaction was considered theoretical when the sources revealed at least one common pathway or transport route subject to inhibition or induction for the CM and the cancer drug combination, but where no study was available to determine its relevance or only *in vitro* studies supported the interaction risk. A potential clinical interaction was retained if the sources identified at least one common pathway or transport route subject to inhibition or induction for the CM and the cancer drug combination, and where animal or human studies supported an interaction risk. A potential clinical interaction where existing human data did not support a significant interaction led to downscaling of the interaction potential (theoretical interaction). The clinical relevance of potential clinical interactions was deemed low if the studies showed or suggested only a mild increase or decrease in cancer drug exposure and where no clear toxicity that was suspected to result from the interaction was reported. The clinical relevance was deemed possibly or likely relevant if the studies showed or suggested a significant increase or decrease (greater than 20%) in cancer drug exposure, or if reported cases of toxicity were suspected to have resulted from the interaction. An additional search was sometimes necessary to deepen the analysis of the clinical relevance of a suspected interaction (e.g. further search for specific metabolic or transporter routes, or search for interactions with other drugs or compounds with similar disposition routes to that of the studied anticancer agent).

### Data analysis

A descriptive analysis was performed for the whole population included and for each subgroup of CM users and non-users. Comparison between groups (CM users versus non-users) was explored by using a non-parametric Wilcoxon’s rank-sum test for continuous variables (age, education level, weight, cancer stage, and number of cancer treatments) and a chi-square test for categorical variables (sex, origin, marital status, health insurance type, cancer type, cancer stage [TNM], and cancer treatment type). Descriptive statistics (median, frequencies) and exploratory comparative statistics were computed with STATA software (version 14, 2015; StataCorp LP, College Station TX, USA). CM users were further divided into users of biologically based CM and users of non-biologically based CM to explore the potential for CM-anticancer agent interactions within our study population from the current literature.

## Supplementary information

Supplementary Data

## Data Availability

Data are available upon request from the authors.
