# Supplementary material for: Complementary medicine use during cancer treatment and potential herb-drug interactions from a cross-sectional study in an academic centre
Source: Sci Rep. 2019 Mar 25;9:5078. doi: 10.1038/s41598-019-41532-3 (PMC6434040; doi:10.1038/s41598-019-41532-3)
Supplement: Supplementary file 1 — Supplementary Data [file 41598_2019_41532_MOESM1_ESM.pdf]

COMPLEMENTARY MEDICINE USE DURING CANCER TREATMENT AND POTENTIAL HERB-DRUG INTERACTIONS FROM A CROSS-SECTIONAL STUDY IN AN ACADEMIC CENTRE.

Mégane Jermini, Julie Dubois, Pierre-Yves Rodondi, Khalil Zaman, Thierry Buclin, Chantal Csajka, Angela Orcurto, Laura Rothuizen

SUPPLEMENTARY INFORMATION

SUPPLEMENTARY QUESTIONNAIRE S1

QUESTIONNAIRE

ETUDE SUR L'UTILISATION DES MÉDECINE COMPLÉMENTAIRE EN ONCOLOGIE

N° d'identifiant pour l'étude (ex. 008): ☐

Date de l'entretien (jj/mm/aa) : ………………….

Dans ce questionnaire, le terme de *médecine complémentaire* est défini comme suit : il s'agit des médecines qui ne sont pas dans la tradition du pays ou qui ne sont pas intégrés dans le système de santé dominant. Cela regroupe donc aussi d'autres termes utilisés comme médecine alternative, douce, parallèle, naturelle ou holistique. Si vous ne savez pas ce qu'est une médecine complémentaire, vous trouverez quelques exemples à la question 12. Dans ce questionnaire, nous nous intéressons à toute forme de prescription de médecine complémentaire, que ce soit par un médecin, un pharmacien, un thérapeute ou un traitement que vous avez pris vous-même sans recommandation d'un professionnel de santé.

Questions

1. Age : ……………
2. Sexe : homme ☐ femme ☐
3. Ethnie :

   Europe ☐
   Afrique ☐
   Amérique centrale et du sud ☐
   Amérique du nord ☐
   Asie ☐
   Océanie ☐
   Inconnu / apatride ☐
   Ne souhaite pas répondre ☐

4. Dernière formation suivie :

   Ecole obligatoire ☐
   Gymnase ☐
   Apprentissage ☐
   Haute école ☐
   Université ☐

5. Etes-vous au bénéfice d'une assurance complémentaire qui couvre des thérapies de médecine complémentaire :

   Oui ☐

Non ☐
Ne sais pas ☐

6. Etat civil :

Célibataire ☐
Marié ou partenariat enregistré ☐
Séparé/divorcé ☐
Ne souhaite pas répondre ☐

7. **Au cours de votre vie**, avez-vous déjà eu recours à une/des médecine(s) complémentaire(s)?

Oui ☐
Non ☐

Si oui, passez à la question 9. Si non, passez à la question 8.

8. Vous n'avez jamais eu recours à des médecines complémentaires parce que (plusieurs réponses possibles) :

Vous n'y croyez pas ☐
C'est trop cher ☐
Vous avez entendu des avis négatifs ☐
Cela ne vous intéresse pas ☐
Vous ne connaissez pas assez bien ce sujet ☐

Autre (spécifier) :
……………………………………………………………………………………………
……………………………………………………………………………………………

Si vous n'avez jamais eu recours à de la médecine complémentaire, nous vous demandons de répondre encore aux questions 21 et 22.

9. **Depuis que votre cancer a été diagnostiqué**, avez-vous eu recours à une/des médecine(s) complémentaire(s) ?

Oui ☐
Non ☐

10. **Actuellement**, avez-vous recours à une/des médecine(s) complémentaire(s) ?

Oui ☐
Non ☐

11. Si oui, laquelle (plusieurs réponses possibles) :

Acupuncture ☐
Médecine traditionnelle chinoise avec herbes chinoises ☐
Médecine ayurvédique ☐
Compléments alimentaires
(oligo-éléments, vitamines et/ou minéraux) ☐
Homéopathie ☐
Réflexologie ☐
Aromathérapie/huiles essentielles ☐
Hypnose ☐
Méditation ☐
Naturopathie ☐
Phytothérapie ☐
Reiki ☐
Yoga ☐
Thé vert ☐
Huile de poisson ☐

Autre (spécifier laquelle) :
……………………………………………………………………………………………………...................
……………………………………………………………………………………………………....................
……………………………………………………………………………………………………..................

12. Si vous prenez un(des) médicament(s) de la médecine complémentaire *ingérées ou injectées*, pouvez-vous mentionner le(s) noms commercial(ux) ou le nom de la/les substance(s), les doses, les dates de prises et les raisons de leur utilisation?

Substance/ Nom commercial	Dose	Période(s)/ Dates de prises	Indication* (1-5)

*Indication : pour quelle raison prenez-vous ce traitement de médecine complémentaire (plusieurs réponses possibles)?

1. Prévention de la rechute
2. Améliorer l'immunité
3. Traiter le cancer
4. Diminuer les effets secondaires de la chimiothérapie
5. Autre (spécifier) :

13. Pour vous, comment agissent les traitements de médecine complémentaire que vous prenez ? *(noter les mots employés par le patient)*
…….
…….
…….
…….

14. Qui vous a recommandé de suivre un traitement de médecines complémentaires pendant le traitement contre le cancer ?

Famille/amis ☐
Médecin de famille ☐
Médecin oncologue ☐
Pharmacien ☐
Thérapeute complémentaire ☐
Médias (journal, radio, télévision, internet,…) ☐
Choix personnel ☐

Autre (spécifier) :
…….
…….

15. Que pensez-vous des affirmations suivantes : Les traitements de médecine complémentaire que je prends sont très efficaces :

tout à fait d'accord ☐ d'accord ☐ ni d'accord, ni pas d'accord ☐ pas d'accord ☐ pas du tout d'accord ☐

16. Les traitements de médecine complémentaire que je prends peuvent avoir des effets secondaires :

tout à fait d'accord ☐ d'accord ☐ ni d'accord, ni pas d'accord ☐ pas d'accord ☐ pas du tout d'accord ☐

Si oui, lesquels redoutez-vous le plus ?
…….
…….

17. Les traitements de médecine complémentaire que je prends peuvent avoir un effet **négatif** sur le traitement du cancer :

tout à fait d'accord ☐ d'accord ☐ ni d'accord, ni pas d'accord ☐ pas d'accord ☐ pas du tout d'accord ☐

Si oui, que craignez-vous le plus ?
…….
…….

18. Les traitements de médecine complémentaire que je prends peuvent avoir un effet **positif** sur le traitement du cancer :

tout à fait d'accord ☐ d'accord ☐ ni d'accord, ni pas d'accord ☐ pas d'accord ☐ pas du tout d'accord ☐

Si oui, quel effet positif principal en attendez-vous ?
…………………………………………………………………………………………………
…………………………………………………………………………………………………

19. Je dispose de suffisamment d'informations sur les **bénéfices** des médecines complémentaires que je prends :

tout à fait d'accord ☐ d'accord ☐ ni d'accord, ni pas d'accord ☐ pas d'accord ☐ pas du tout d'accord ☐

20. Je dispose de suffisamment d'informations sur les **risques** des médecines complémentaires que je prends :

tout à fait d'accord ☐ d'accord ☐ ni d'accord, ni pas d'accord ☐ pas d'accord ☐ pas du tout d'accord ☐

21. Est-ce qu'il vous a été demandé si vous utilisez une médecine complémentaire

Par votre médecin oncologue ?	Par un infirmier/infirmière en oncologie ?
Oui ☐	Oui ☐
Non ☐	Non ☐

22. Est-ce qu'il vous a été proposé d'utiliser une médecine complémentaire

Par votre médecin oncologue ?	Par un infirmier/infirmière en oncologie ?
Oui ☐	Oui ☐
Non ☐	Non ☐

Si oui, laquelle/ lesquelles :
………………………………………………………………………………………
………………………………………………………………………………………

23. Avez-vous dit que vous utilisez une médecine complémentaire

A votre médecin oncologue ?	A un infirmier/infirmière en oncologie ?
Oui ☐	Oui ☐
Non ☐	Non ☐

24. Si oui la question 23, votre médecin oncologue/ infirmier/ère connaissait-il/elle cette thérapie ?

Oui ☐
Non ☐
Ne sais pas ☐

25. Si oui à la question 23, comment a-t-il/elle répondu :

Encouragé à continuer le traitement de médecine complémentaire ☐
Demandé d'arrêter le traitement de médecine complémentaire ☐
N'a pas fait de recommandation ☐

Autre (spécifier) :
……………………………………………………………………………………………
……………………………………………………………………………………………

26. Si non à la question 23, pourquoi n'en avez-vous pas parlé :

Parce qu'il/elle ne me l'a pas demandé ☐
Il/elle ne comprendrait pas ☐
Il/elle ne serait pas content ☐
J'ai oublié de le mentionner ☐
Cela n'a pas d'importance de le dire ☐

Autre (spécifier) :
……………………………………………………………………………………………
……………………………………………………………………………………………

27. Si votre médecin oncologue jugeait inapproprié de continuer la prise concomitante de médecines complémentaires en même temps que le traitement anticancéreux, continueriez-vous à les utiliser ?

Oui, dans tous les cas ☐
Non, j'arrêterais sans problème ☐
J'aurais besoin de justifications avant de décider ☐
Je n'arrêterais que si les risques de continuer sont graves ☐

Souhaitez-vous préciser votre réponse :

……………………………………………………………………………………………
……………………………………………………………………………………………
……………………………………………………………………………………………
……………………………………………………………………………………………

ANNEXE 2 : **DONNÉES MÉDICALES DU PATIENT**

N° d'identifiant pour l'étude (ex. 008): ☐

Age : ....

Sexe : homme ☐ femme ☐

Poids (kg) : ……

*Insuffisance rénale actuelle ? oui ☐ non ☐ ? ☐

*Insuffisance ou perturbation actuelle des tests hépatiques? oui ☐ non ☐ ?☐

* Oui : si figure dans la liste des diagnostics récents (<1an) selon le dossier médical
Non : si fonction organique précisée comme normale dans le dossier médical (< 1an)
? : si aucune précision n'est donnée dans le dossier médical ou que la dernière évaluation des fonctions organiques date de >1 an

Type de cancer:

- Nez ☐
- Gorge ou oreille ☐
- Cerveau ☐
- Sein ☐
- Estomac ☐
- Intestin ☐
- Peau ☐
- Poumons ☐
- Ovaires ☐
- Œsophage ☐
- Leucémie ☐
- Lymphome ☐
- Utérus col ou endomètre ☐
- Rein ☐
- Vessie ☐
- Prostate ☐
- Pancréas ☐
- Myélome ☐
- Foie ☐

Autre, lequel :
…………………..

Précisions diagnostiques (type, stade, etc.)

……
……

Année de diagnostic du cancer : ....

Traitement(s) reçus (plusieurs réponses possibles):

- Chirurgie ☐
- Chimiothérapie traditionnelle (cycles) ☐
- Traitement anticancéreux ciblé ☐
- Radiothérapie ☐
- Traitement hormonal/ préventif ☐

Autre, lequel :
……………………………………………………………………………………………

Traitements **actuellement** en cours (préciser ensuite dans la table):

- Chirurgie ☐
- Chimiothérapie traditionnelle (cycles) ☐
- Traitement anticancéreux ciblé ☐
- Radiothérapie ☐
- Traitement hormonal/ préventif ☐

Autre, lequel :
……………………………………………………………………………………………

Traitement(s) anticancéreux en cours :

**Substance (DCI)**	**Dose**	**Voie d'admin.**	**Date Initiation**	**Dernière dose (DD)/ Prochaine dose (PD)**	**Date d'arrêt***
Ex. tamoxifène	*20 mg 1x/j*	*po*	*Avril 2012*	*En cours*	*?*

*si connu

Eventuelles remarques ou autres données spécifiques du dossier dont l'utilité est en lien direct avec les objectifs du projet :

Exemples:

-Notes médicales/ infirmières relatives à des informations quant l'utilisation de médecines complémentaires rapportées à l'équipe soignante

-Demande de consultation auprès de la division de pharmacologie clinique pour évaluation du risque potentiel d'interaction. Impact éventuel sur le choix/ la dose des prescriptions
d'anticancéreux …………………………………………………………………………
…………………………………………………………………………………………
…………………………………………………………………………………………
…………………………………………………………………………………………
…………………………………………………………………………………………
…………………………………………………………………………………………
…………………………………………………………………………………………
…………………………………………………………………………………………
…………………………………………………………………………………………
…………………………………………………………………………………………
…………………………………………………………………………………………
…………………………………………………………………………………………
…………………………………………………………………………………………
…………………………………………………………………………………………
…………………………………………………………………………………………
…………………………………………………………………………………………
…………………………………………………………………………………………
…………………………………………………………………………………………
…………………………………………………………………………………………
…………………………………………………………………………………………
…………………………………………………………………………………………
…………………………………………………………………………………………
……………………

SUPPLEMENTARY QUESTIONNAIRE S2

QUESTIONNAIRE

STUDY ON THE USE OF COMPLEMENTARY MEDICINE IN ONCOLOGY

Study identification N° (e.g. 008): ☐

Interview date (DD/MM/YY): …………………

In this questionnaire, the term *complementary medicine* is defined as a broad set of healthcare practices that are not part of a country's own tradition or conventional medicine and are not fully integrated into the dominant healthcare system. This definition is inclusive of other terms such as alternative, parallel, natural or holistic medicine. If you do not know about complementary medicine, some examples can be found under question 12. We are interested in all types of complementary medicine, whether prescribed by a physician, a pharmacist or a therapist, or whether your use them on your own initiative, outside of any recommendation from a healthcare professional.

Questions

28. Age: ……………

29. Sex: male ☐ female ☐

30. Origin:

    Europe ☐
    Africa ☐
    Central/South America ☐
    North America ☐
    Asia ☐
    Oceania ☐
    Unknown/stateless ☐
    Unknown (not answered) ☐

31. Education level:

    Primary ☐
    Secondary ☐
    Apprenticeship ☐
    High school, college ☐
    University ☐

32. Do you have additional coverage for complementary medicine included in your health insurance?

    Yes ☐
    No ☐
    I don't know ☐

33. Marital status:

Single ☐
Married/civil partnership/widowed ☐
Divorced/separated ☐
Unknown (not answered) ☐

34. Have you **at any time in your life** used complementary medicine?

Yes ☐
No ☐

If yes, go to question 9. If no, go to question 8.

35. You have never used complementary medicine because (several answers are possible):

You do not believe in it ☐
It is too expensive ☐
You have heard bad comments about it ☐
You are not interested in it ☐
You do not know enough about it ☐

Other (specify):
………………………………………………………………………………………………
………………………………………………………………………………………………
……………….

If you have never used complementary medicine, please go on to answer questions 21 and 22 only.

36. Have you used complementary medicine **since your cancer was diagnosed?**

Yes ☐
No ☐

37. Are you **currently** using complementary medicine?

Yes ☐
No ☐

38. If yes, what complementary medicine(s) are you using? (several answers are possible):

Acupuncture ☐
Traditional Chinese medicine (Chinese herbal remedies) ☐
Ayurveda ☐
Dietary supplements
(trace elements, vitamins and/or minerals) ☐
Homeopathy ☐
Reflexology ☐
Aromatherapy/essential oils ☐
Hypnosis ☐
Meditation ☐
Naturopathy ☐
Herbal remedies ☐
Reiki ☐
Yoga ☐
Green tea ☐
Fish oil ☐

Other (please specify):
……………………………………………………………………………………..................
……………………………………………………………………………………..................
……………………………………………………………………………………..................

39. If you are taking one or several *oral (ingested) or injected* complementary medicine(s), could you mention the trade name(s) or substance(s), the dose(s), and the date(s) at which it/they was/were taken, and the reason for using it/them ?

Substance/ Trade name	Dose	Period(s)/ Dates of use	Indication* (1-5)

*Indication: For what reason are you taking this/these complementary medicine(s)? (several answers are possible)

1. To prevent relapse
2. To improve immune system
3. To treat cancer
4. To attenuate adverse effects of chemotherapy
5. Other (specify):

40. According to you, how does/do the complementary medicine(s) you take exert its/their effect *(reporting in words as expressed by the patient)*
………………………………………………………………………………………….....................
………………………………………………………………………………………….................
………………………………………………………………………………………….....................
………………………………………………………………………………………….................

41. Who recommended that you use complementary medicine(s) during cancer treatment?

Family/friends ☐
Family physician ☐
Oncologist ☐
Pharmacist ☐
Complementary medicine therapist ☐
Media (journals, radio, television, Internet) ☐
Personal choice ☐

Other (please specify):
……
……

What is your opinion about the following statements?

42. The complementary medicine(s) I take is/are very effective.

strongly agree ☐ agree ☐ neither agree nor disagree ☐ disagree ☐ strongly disagree ☐

43. The complementary medicine(s) I take can have side effects.

strongly agree ☐ agree ☐ neither agree nor disagree ☐ disagree ☐ strongly disagree ☐

If yes, which side effects do you most fear?
………………………………………………………………………………………….....................
………………………………………………………………………………………….................

44. The complementary medicine(s) I take can have a **negative** effect on the cancer treatment.

strongly agree ☐ agree ☐ neither agree nor disagree ☐ disagree ☐ strongly disagree ☐

If yes, which negative effect do you most fear?
………………………………………………………………………………………….....................
………………………………………………………………………………………….................

45. The complementary medicine(s) I take can have a **positive** effect on the cancer treatment.

strongly agree ☐ agree ☐ neither agree nor disagree ☐ disagree ☐ strongly disagree ☐

If yes, which main positive effect are you expecting?
………………………………………………………………………………………………..................
………………………………………………………………………………………………..................

46. I am sufficiently informed about the **benefits** of the complementary medicine(s) that I take.

strongly agree ☐ agree ☐ neither agree nor disagree ☐ disagree ☐ strongly disagree ☐

47. I am sufficiently informed about the **risks** of the complementary medicine(s) that I take.

strongly agree ☐ agree ☐ neither agree nor disagree ☐ disagree ☐ strongly disagree ☐

48. Were you asked if you use complementary medicine(s):

By your oncologist? By a nurse in oncology?

Yes ☐ Yes ☐
No ☐ No ☐

49. Was the use of complementary medicine(s) offered:

By your oncologist? By a nurse in oncology?

Yes ☐ Yes ☐
No ☐ Non ☐

If yes, what complementary medicine(s)?
…………………………………………………………………………………………
…………………………………………………………………………………………

50. Did you mention the use of complementary medicine(s):

To your oncologist? To a nurse in oncology?

Yes ☐ Yes ☐
No ☐ No ☐

51. If the answer to question 23 is yes, was your oncologist/the nurse in oncology familiar with this therapy?

Yes ☐
No ☐
I don't know ☐

52. If the answer to question 23 is yes, how did he/she react?

He/she encouraged you to continue complementary medicine(s) ☐
He/she requested you stop using complementary medicine(s) ☐
He/she made no recommendation ☐

Other (please specify):
……………………………………………………………………………………………
……………………………………………………………………………………………

53. If the answer to question 23 is no, why did you not mention using complementary medicine(s)?

He/she did not ask me ☐
He/she would not understand ☐
He/she would not be happy about it ☐
I forgot to mention it ☐
It is not important to mention it ☐

Other (please specify):
……………………………………………………………………………………………
……………………………………………………………………………………………

54. If your oncologist deems it inappropriate to continue taking complementary medicine(s) concomitantly with your cancer treatment, would you go on taking it/them?

Yes, in all cases ☐
No, I would easily stop taking it/them ☐
I would need justifications before I decide whether to stop ☐
I would only stop if there are serious risks of pursuing it/them ☐

If you wish to add further detail to your answer:

……………………………………………………………………………………………
……………………………………………………………………………………………
……………………………………………………………………………………………
……………………………………………………………………………………………

APPENDIX 2: **PATIENT MEDICAL DATA**

Study identification N° (e.g. 008): ☐

Age: ....

Sex: male ☐ female ☐

Weight (kg): ……

*Current renal function impairment? yes ☐ no ☐ ? ☐

*Current liver function impairment or elevated liver tests? yes ☐ no ☐ ?☐

* Yes: if mentioned in a recent list of diagnoses (<1 year) in the patient's medical record.
No: if organ function was recently (<1 year) specified as normal in the patient's medical record.
?: if no detailed information exists in the medical record, or if the last assessment of organ functions was >1 year ago.

Type of cancer:

- Nose ☐
- Ear and throat ☐
- Brain ☐
- Breast ☐
- Stomach ☐
- Intestine ☐
- Skin ☐
- Lung ☐
- Ovary ☐
- Oesophagus ☐
- Leukaemia ☐
- Lymphoma ☐
- Uterus, cervix or endometrium ☐
- Kidney ☐
- Bladder ☐
- Prostate ☐
- Pancreas ☐
- Myeloma ☐
- Liver ☐

Other, specified:
………………………..

Diagnostic details (histology type, staging, etc.)

………………………………………………………………………………………………
………………………………………………………………………………………………

Year of cancer diagnosis: ....

Treatment(s) dispensed (several answers possible):

- Surgery ☐
- Conventional chemotherapy (cycles) ☐
- Targeted cancer therapy ☐
- Radiotherapy ☐
- Hormonal treatment/preventive therapy ☐

Other, specified:
………………………………………………………………………………………

Currently ongoing cancer therapy (detail in table below):

- Surgery ☐
- Conventional chemotherapy (cycles) ☐
- Targeted cancer therapy ☐
- Radiotherapy ☐
- Hormonal treatment/preventive therapy ☐

Other, specified:
………………………………………………………………………………………

Current ongoing cancer treatment(s):

**Substance (DCI)**	**Dose**	**Route of administration**	**Initiation date**	**Last dose (LD)/next dose (ND)**	**Date stopped***
e.g. tamoxifen	*20 mg 1x/j*	*po*	*April 2012*	*Ongoing*	*?*

*if known

Any other comment or data from the patient's medical record identified as of interest to this study?

For example:

- medical or nursing staff notes about complementary medicine use reported to the caring team

- Expert advice from clinical pharmacology regarding interaction risk evaluation and possible impact on the choice of cancer treatment or dose, etc.

……………………………………………………………………………………………
……………………………………………………………………………………………
……………………………………………………………………………………………
……………………………………………………………………………………………
……………………………………………………………………………………………
……………………………………………………………………………………………
……………………………………………………………………………………………
……………………………………………………………………………………………
……………………………………………………………………………………………
……………………………………………………………………………………………
……………………………………………………………………………………………
……………………………………………………………………………………………
……………………………………………………………………………………………
……………………………………………………………………………………………
……………………………………………………………………………………………
……………………………………………………………………………………………
……………………………………………………………………………………………
……………………………………………………………………………………………
……………………………………………………………………………………………
……………………………………………………………………………………………
……………………………………………………………………………………………
……………………………………………………………………………………………

SUPPLEMENTARY DATA S3: ARTICLES SELECTED TO ELABORATE THE TABLE OF POTENTIAL PHARMACOKINETIC INTERACTIONS BETWEEN HERBAL MEDICINES AND ANTINEOPLASTIC AGENTS

A) Articles discussing CM substances recurrently cited as presenting documented or theoretical potential for interaction

1. Ali-Shtayeh MS, Jamous RM, Salameh NM, Jamous RM, Hamadeh AM. Complementary and alternative medicine use among cancer patients in Palestine with special reference to safety-related concerns. J Ethnopharmacol. 2016 Jul 1;187:104-22. PubMed PMID: 27125594.
2. Alsanad SM, Howard RL, Williamson EM. An assessment of the impact of herb-drug combinations used by cancer patients. BMC Complement Altern Med. 2016 Oct 18;16(1):393. PubMed PMID: 27756298; PubMed Central PMCID: PMCPMC5070090.
3. Alsanad SM, Williamson EM, Howard RL. Cancer patients at risk of herb/food supplement-drug interactions: A systematic review. Phytother Res. 2014 Dec;28(12):1749-55. PubMed PMID: 25158128.
4. Andersen MR, Sweet E, Lowe KA, Standish LJ, Drescher CW, Goff BA. Dangerous combinations: Ingestible cam supplement use during chemotherapy in patients with ovarian cancer. J Altern Complement Med. 2013 Aug;19(8):714-20. PubMed PMID: 23445210; PubMed Central PMCID: PMCPMC3731675.
5. Awortwe C, Makiwane M, Reuter H, Muller C, Louw J, Rosenkranz B. Critical evaluation of causality assessment of herb-drug interactions in patients. Br J Clin Pharmacol. 2018 Apr;84(4):679-93. PubMed PMID: 29363155; PubMed Central PMCID: PMCPMC5867089.
6. Ben-Arye E, Lavie O, Samuels N, et al. Safety of herbal medicine use during chemotherapy in patients with ovarian cancer: A "bedside-to-bench" approach. Med Oncol. 2017 Apr;34(4):54. PubMed PMID: 28238155.
7. Ben-Arye E, Samuels N, Goldstein LH, et al. Potential risks associated with traditional herbal medicine use in cancer care: A study of middle eastern oncology health care professionals. Cancer. 2016 Feb 15;122(4):598-610. PubMed PMID: 26599199.
8. Berretta M, Della Pepa C, Tralongo P, et al. Use of complementary and alternative medicine (cam) in cancer patients: An italian multicenter survey. Oncotarget. 2017 Apr 11;8(15):24401-14. PubMed PMID: 28212560; PubMed Central PMCID: PMCPMC5421857.
9. Bossaer JB, Odle BL. Probable etoposide interaction with echinacea. J Diet Suppl. 2012 Jun;9(2):90-5. PubMed PMID: 22607644.
10. Carcelero E, Anglada H, Tuset M, Creus N. Interactions between oral antineoplastic agents and concomitant medication: A systematic review. Expert Opin Drug Saf. 2013 May;12(3):403-20. PubMed PMID: 23586848.

11. Collado-Borrell R, Escudero-Vilaplana V, Romero-Jimenez R, Iglesias-Peinado I, Herranz-Alonso A, Sanjurjo-Saez M. Oral antineoplastic agent interactions with medicinal plants and food: An issue to take into account. J Cancer Res Clin Oncol. 2016 Nov;142(11):2319-30. PubMed PMID: 27316629.

12. De Smet PA. Health risks of herbal remedies: An update. Clin Pharmacol Ther. 2004 Jul;76(1):1-17. PubMed PMID: 15229459.

13. Ernst E. The risk–benefit profile of commonly used herbal therapies: Ginkgo, st. John's wort, ginseng, echinacea, saw palmetto, and kava. Annals of Internal Medicine. 2002;136(1):42-53.

14. Firkins R, Eisfeld H, Keinki C, et al. The use of complementary and alternative medicine by patients in routine care and the risk of interactions. J Cancer Res Clin Oncol. 2018 Mar;144(3):551-7. PubMed PMID: 29356888.

15. Fujita K-i. Cytochrome p450 and anticancer drugs. Current Drug Metabolism. 2006;7(1):23-37.

16. Goey AK, Mooiman KD, Beijnen JH, Schellens JH, Meijerman I. Relevance of in vitro and clinical data for predicting cyp3a4-mediated herb-drug interactions in cancer patients. Cancer Treat Rev. 2013 Nov;39(7):773-83. PubMed PMID: 23394826.

17. Gurley BJ. Pharmacokinetic herb-drug interactions (part 1): Origins, mechanisms, and the impact of botanical dietary supplements. Planta Med. 2012 Sep;78(13):1478-89. PubMed PMID: 22322396.

18. Haefeli WE, Carls A. Drug interactions with phytotherapeutics in oncology. Expert Opin Drug Metab Toxicol. 2014 Mar;10(3):359-77. PubMed PMID: 24387348.

19. HemaIswarya S, Doble M. Potential synergism of natural products in the treatment of cancer. Phytother Res. 2006 Apr;20(4):239-49. PubMed PMID: 16557604.

20. Hermann R, von Richter O. Clinical evidence of herbal drugs as perpetrators of pharmacokinetic drug interactions. Planta Med. 2012 Sep;78(13):1458-77. PubMed PMID: 22855269.

21. Hierl M, Pfirstinger J, Andreesen R, et al. Complementary and alternative medicine: A clinical study in 1,016 hematology/oncology patients. Oncology. 2017;93(3):157-63. PubMed PMID: 28641281.

22. Hubner J, Hanf V. Commonly used methods of complementary medicine in the treatment of breast cancer. Breast Care (Basel). 2013 Oct;8(5):341-7. PubMed PMID: 24415987; PubMed Central PMCID: PMCPMC3861850.

23. Izzo AA, Ernst E. Interactions between herbal medicines and prescribed drugs: An updated systematic review. Drugs. 2009 2009/09/01;69(13):1777-98. PubMed PMID: 19719333.

24. Kivisto KT, Kroemer HK, Eichelbaum M. The role of human cytochrome p450 enzymes in the metabolism of anticancer agents: Implications for drug interactions. Br J Clin Pharmacol. 1995 Dec;40(6):523-30. PubMed PMID: 8703657; PubMed Central PMCID: PMCPMC1365206.

25. Lee RT, Barbo A, Lopez G, et al. National survey of us oncologists' knowledge, attitudes, and practice patterns regarding herb and supplement use by patients with cancer. J Clin Oncol. 2014 Dec 20;32(36):4095-101. PubMed PMID: 25403205; PubMed Central PMCID: PMCPMC4517940.

26. Loquai C, Schmidtmann I, Garzarolli M, et al. Interactions from complementary and alternative medicine in patients with melanoma. Melanoma Res. 2017 Jun;27(3):238-42. PubMed PMID: 28252553.

27. McCune JS, Hatfield AJ, Blackburn AA, Leith PO, Livingston RB, Ellis GK. Potential of chemotherapy-herb interactions in adult cancer patients. Support Care Cancer. 2004 Jun;12(6):454-62. PubMed PMID: 14991387.

28. Meijerman I, Beijnen JH, Schellens JH. Herb-drug interactions in oncology: Focus on mechanisms of induction. Oncologist. 2006 Jul-Aug;11(7):742-52. PubMed PMID: 16880233.

29. Olaku O, White JD. Herbal therapy use by cancer patients: A literature review on case reports. Eur J Cancer. 2011 Mar;47(4):508-14. PubMed PMID: 21185719; PubMed Central PMCID: PMCPMC3057114.

30. Pourroy B, Letellier C, Helvig A, Chanet B, De Crozals F, Alessandra C. Development of a rapid risk evaluation tool for herbs/drugs interactions in cancer patients: A multicentric experience in south of france. Eur J Cancer Care (Engl). 2017 Nov;26(6):e12752-n/a. PubMed PMID: 28881077.

31. Ramos-Esquivel A, Víquez-Jaikel Á, Fernández C. Potential drug-drug and herb-drug interactions in patients with cancer: A prospective study of medication surveillance. Journal of Oncology Practice. 2017 2017/07/01;13(7):e613-e22.

32. Shi S, Klotz U. Drug interactions with herbal medicines. Clin Pharmacokinet. 2012 Feb 1;51(2):77-104. PubMed PMID: 22257149.

33. Sparreboom A, Cox MC, Acharya MR, Figg WD. Herbal remedies in the united states: Potential adverse interactions with anticancer agents. J Clin Oncol. 2004 Jun 15;22(12):2489-503. PubMed PMID: 15197212.

34. Spinella M. The importance of pharmacological synergy in psychoactive herbal medicines. Altern Med Rev. 2002 Apr;7(2):130-7. PubMed PMID: 11991792.

35. Treasure J. Herbal medicine and cancer: An introductory overview. Semin Oncol Nurs. 2005 Aug;21(3):177-83. PubMed PMID: 16092805.

36. Tsai HH, Lin HW, Simon Pickard A, Tsai HY, Mahady GB. Evaluation of documented drug interactions and contraindications associated with herbs and dietary supplements: A systematic literature review. Int J Clin Pract. 2012 Nov;66(11):1056-78. PubMed PMID: 23067030.

37. Undevia SD, Gomez-Abuin G, Ratain MJ. Pharmacokinetic variability of anticancer agents. Nat Rev Cancer. 2005 Jun;5(6):447-58. PubMed PMID: 15928675.

38. Unger M. Pharmacokinetic drug interactions involving ginkgo biloba. Drug Metab Rev. 2013 Aug;45(3):353-85. PubMed PMID: 23865865.

39. van Erp NP, Baker SD, Zhao M, et al. Effect of milk thistle (silybum marianum) on the pharmacokinetics of irinotecan. Clin Cancer Res. 2005 Nov 1;11(21):7800-6. PubMed PMID: 16278402.

40. Wang CZ, Calway T, Yuan CS. Herbal medicines as adjuvants for cancer therapeutics. Am J Chin Med. 2012;40(4):657-69. PubMed PMID: 22809022; PubMed Central PMCID: PMCPMC3415888.

41. Werneke U, Earl J, Seydel C, Horn O, Crichton P, Fannon D. Potential health risks of complementary alternative medicines in cancer patients. Br J Cancer. 2004 Jan 26;90(2):408-13. PubMed PMID: 14735185; PubMed Central PMCID: PMCPMC2410154.

42. Yasueda A, Urushima H, Ito T. Efficacy and interaction of antioxidant supplements as adjuvant therapy in cancer treatment: A systematic review. Integr Cancer Ther. 2016 Mar;15(1):17-39. PubMed PMID: 26503419; PubMed Central PMCID: PMCPMC5736082.

43. Zeller T, Muenstedt K, Stoll C, et al. Potential interactions of complementary and alternative medicine with cancer therapy in outpatients with gynecological cancer in a comprehensive cancer center. J Cancer Res Clin Oncol. 2013 Mar;139(3):357-65. PubMed PMID: 23099993.

44. Zhou S, Gao Y, Jiang W, Huang M, Xu A, Paxton JW. Interactions of herbs with cytochrome p450. Drug Metab Rev. 2003 Feb;35(1):35-98. PubMed PMID: 12635815.

B) Articles used for the assessment of potential two-by-two herb-drug interactions in our patients

1. Ahn KH, Yi KW, Park HT, et al. Anti-proliferative effect of klimaktoplan(r) on human breast cancer cells. Arch Gynecol Obstet. 2013 Oct;288(4):833-8. PubMed PMID: 23595584.

2. Braunlich M, Christensen H, Johannesen S, et al. In vitro inhibition of cytochrome p450 3a4 by aronia melanocarpa constituents. Planta Med. 2013 Jan;79(2):137-41. PubMed PMID: 23250807.

3. Braunlich M, Slimestad R, Wangensteen H, Brede C, Malterud KE, Barsett H. Extracts, anthocyanins and procyanidins from aronia melanocarpa as radical scavengers and enzyme inhibitors. Nutrients. 2013 Mar 4;5(3):663-78. PubMed PMID: 23459328; PubMed Central PMCID: PMCPMC3705312.

4. Budzinski JW, Foster BC, Vandenhoek S, Arnason JT. An in vitro evaluation of human cytochrome p450 3a4 inhibition by selected commercial herbal extracts and tinctures. Phytomedicine. 2000 Jul;7(4):273-82. PubMed PMID: 10969720.

5. Celik I, Isik I. Determination of chemopreventive role of foeniculum vulgare and salvia officinalis infusion on trichloroacetic acid-induced increased serum marker enzymes lipid

peroxidation and antioxidative defense systems in rats. Nat Prod Res. 2008 Jan 10;22(1):66-75. PubMed PMID: 17999340.

6. Chamorro-Cevallos G, Garduno-Siciliano L, Barron BL, Madrigal-Bujaidar E, Cruz-Vega DE, Pages N. Chemoprotective effect of spirulina (arthrospira) against cyclophosphamide-induced mutagenicity in mice. Food Chem Toxicol. 2008 Feb;46(2):567-74. PubMed PMID: 17928122.

7. Chen J, Hui E, Ip T, Thompson LU. Dietary flaxseed enhances the inhibitory effect of tamoxifen on the growth of estrogen-dependent human breast cancer (mcf-7) in nude mice. Clin Cancer Res. 2004 Nov 15;10(22):7703-11. PubMed PMID: 15570004.

8. Dresser GK, Wacher V, Wong S, Wong HT, Bailey DG. Evaluation of peppermint oil and ascorbyl palmitate as inhibitors of cytochrome p4503a4 activity in vitro and in vivo. Clin Pharmacol Ther. 2002 Sep;72(3):247-55. PubMed PMID: 12235445.

9. Egashira K, Sasaki H, Higuchi S, Ieiri I. Food-drug interaction of tacrolimus with pomelo, ginger, and turmeric juice in rats. Drug Metab Pharmacokinet. 2012;27(2):242-7. PubMed PMID: 22123127.

10. Engdal S, Nilsen OG. In vitro inhibition of cyp3a4 by herbal remedies frequently used by cancer patients. Phytother Res. 2009 Jul;23(7):906-12. PubMed PMID: 19170155.

11. Frank A, Unger M. Analysis of frankincense from various boswellia species with inhibitory activity on human drug metabolising cytochrome p450 enzymes using liquid chromatography mass spectrometry after automated on-line extraction. J Chromatogr A. 2006 Apr 21;1112(1-2):255-62. PubMed PMID: 16364338.

12. Ganzera M, Schneider P, Stuppner H. Inhibitory effects of the essential oil of chamomile (matricaria recutita l.) and its major constituents on human cytochrome p450 enzymes. Life Sci. 2006 Jan 18;78(8):856-61. PubMed PMID: 16137701.

13. Karadeniz A, Simsek N, Karakus E, et al. Royal jelly modulates oxidative stress and apoptosis in liver and kidneys of rats treated with cisplatin. Oxid Med Cell Longev. 2011;2011:981793. PubMed PMID: 21904651; PubMed Central PMCID: PMCPMC3166579.

14. Khan M, Shobha JC, Mohan IK, et al. Protective effect of spirulina against doxorubicin-induced cardiotoxicity. Phytother Res. 2005 Dec;19(12):1030-7. PubMed PMID: 16372368.

15. Kim IS, Kim SY, Yoo HH. Effects of an aqueous-ethanolic extract of ginger on cytochrome p450 enzyme-mediated drug metabolism. Pharmazie. 2012 Dec;67(12):1007-9. PubMed PMID: 23346764.

16. Leung H, Hung A, Hui AC, Chan TY. Warfarin overdose due to the possible effects of lycium barbarum l. Food Chem Toxicol. 2008 May;46(5):1860-2. PubMed PMID: 18281140.

17. Li J, Deng Y, Yuan C, et al. Antioxidant and quinone reductase-inducing constituents of black chokeberry (aronia melanocarpa) fruits. J Agric Food Chem. 2012 Nov 21;60(46):11551-9. PubMed PMID: 23131110.

18. Liu Q, Li Y, Hu L, Wang D. Lycium barbarum polysaccharides attenuate cisplatin-induced hair cell loss in rat cochlear organotypic cultures. Int J Mol Sci. 2011;12(12):8982-92. PubMed PMID: 22272115; PubMed Central PMCID: PMCPMC3257112.

19. Maliakal PP, Wanwimolruk S. Effect of herbal teas on hepatic drug metabolizing enzymes in rats. Journal of Pharmacy and Pharmacology. 2001 2001/10/01;53(10):1323-9.

20. Mansky PJ, Wallerstedt DB, Sannes TS, et al. Nccam/nci phase 1 study of mistletoe extract and gemcitabine in patients with advanced solid tumors. Evid Based Complement Alternat Med. 2013;2013:964592. PubMed PMID: 24285980; PubMed Central PMCID: PMCPMC3826324.

21. Mason JK, Fu MH, Chen J, Yu Z, Thompson LU. Dietary flaxseed-trastuzumab interactive effects on the growth of her2-overexpressing human breast tumors (bt-474). Nutr Cancer. 2013;65(3):451-9. PubMed PMID: 23530645.

22. Mishima S, Suzuki KM, Isohama Y, et al. Royal jelly has estrogenic effects in vitro and in vivo. J Ethnopharmacol. 2005 Oct 3;101(1-3):215-20. PubMed PMID: 15946813.

23. Mohan IK, Khan M, Shobha JC, et al. Protection against cisplatin-induced nephrotoxicity by spirulina in rats. Cancer Chemother Pharmacol. 2006 Dec;58(6):802-8. PubMed PMID: 16552571.

24. Saggar JK, Chen J, Corey P, Thompson LU. Dietary flaxseed lignan or oil combined with tamoxifen treatment affects mcf-7 tumor growth through estrogen receptor- and growth factor-signaling pathways. Mol Nutr Food Res. 2010 Mar;54(3):415-25. PubMed PMID: 19904759.

25. Savranoglu S, Tumer TB. Inhibitory effects of spirulina platensis on carcinogen-activating cytochrome p450 isozymes and potential for drug interactions. Int J Toxicol. 2013 Sep-Oct;32(5):376-84. PubMed PMID: 24082030.

26. Steele ML, Axtner J, Happe A, Kroz M, Matthes H, Schad F. Adverse drug reactions and expected effects to therapy with subcutaneous mistletoe extracts (viscum album l.) in cancer patients. Evid Based Complement Alternat Med. 2014;2014:724258. PubMed PMID: 24672577; PubMed Central PMCID: PMCPMC3929984.

27. Subehan, Usia T, Iwata H, Kadota S, Tezuka Y. Mechanism-based inhibition of cyp3a4 and cyp2d6 by indonesian medicinal plants. J Ethnopharmacol. 2006 May 24;105(3):449-55. PubMed PMID: 16414224.

28. Subehan, Zaidi SF, Kadota S, Tezuka Y. Inhibition on human liver cytochrome p450 3a4 by constituents of fennel (foeniculum vulgare): Identification and characterization of a mechanism-based inactivator. J Agric Food Chem. 2007 Dec 12;55(25):10162-7. PubMed PMID: 17988092.

29. Valcheva-Kuzmanova SV, Beronova AB, Momekov GT. Protective effect of aronia melanocarpa fruit juice in a model of cisplatin-induced cytotoxicity in vitro. Folia Med (Plovdiv). 2013 Jul-Dec;55(3-4):76-9. PubMed PMID: 24712286.

30. Weissenstein U, Kunz M, Urech K, Baumgartner S. Interaction of standardized mistletoe (viscum album) extracts with chemotherapeutic drugs regarding cytostatic and cytotoxic effects in

vitro. BMC Complement Altern Med. 2014 Jan 8;14:6. PubMed PMID: 24397864; PubMed Central PMCID: PMCPMC3893555.

31. Yun J, Tomida A, Nagata K, Tsuruo T. Glucose-regulated stresses confer resistance to vp-16 in human cancer cells through a decreased expression of DNA topoisomerase ii. Oncol Res. 1995;7(12):583-90. PubMed PMID: 8704275.

SUPPLEMENTARY TABLE S4 : REFERENCES USED TO ASSESS THE INTERACTION POTENTIAL OF EACH COMPLEMENTARY MEDICINE PRODUCT

**Complementary Medicine**	**References***
Alium Sativum	1A, 2A, 3A, 4A, 11A, 16A, 18A, 20A, 23A, 28A, 30A, 31A, 32A, 33A, 44A, 10B
Aronia Melanocarpia	30A, 2B, 3B, 17B, 29B
Boswellia spp.	11B
Camelia sinensis	1A, 4A, 5A, 7A, 8A, 11A, 19A, 29A, 30A, 31A, 10B
Cimicifuga racemosa	27A, 33A, 1B
Citrus spp.	8A, 11A ,12A, 35A
Curcuma longa	1A, 5A, 6A, 7A, 8A, 19A, 28A, 30A, 9B
Echinacea spp.	3A, 5A, 8A, 9A, 11A, 13A, 18A, 23A, 28A, 31A, 32A, 33A, 4B
Foeniculum vulgare	1A, 7A, 5B, 28B
Gingko biloba	2A, 3A, 4A, 5A, 7A, 8A, 18A, 20A, 28A, 30A, 31A, 33A, 36A, 38A
Glycyrrhiza glabra	30A, 4B
Glycine max	33A
Hydrastis canadensis	3A, 20A, 32A, 41A, 4B
Hypericum perforatum	2A, 3A, 5A, 7A, 8A, 11A, 13A, 16A, 17A, 18A, 19A, 23A, 27A, 28A, 30A, 32A, 33A, 34A, 36A, 37A, 40A, 44A, 4B
Lycium spp.	5A, 16B, 18B
Matricaria recutita	1A, 5A, 11A, 31A, 4B, 12B, 19B
Mentha piperita	5A, 8B, 19B
Oenothera biennis	15A, 23A, 33A
Panax spp	7A, 11A, 13A, 16A, 18A, 20A, 23A, 27A, 28A, 30A, 32A, 33A, 40A
Piper methysticum	13A, 28A, 32A, 34A
Piper nigrum	27B
Serenoa repens	11A, 13A, 23A, 32A, 33A
Sylibum marianum	3A, 7A, 8A, 16A, 20A, 30A, 32A, 33A, 39A, 44A, 41A, 49A
Valeriana officinalis	3A, 7A, 11A, 31A, 33A, 34A, 40A
Viscum album	3A, 6A, 7A, 14A, 22A, 26A, 29A, 43A, 10B, 20B, 26B, 30B
Vitis vinifera	7A, 31A
Zingiber officinalis	4A, 6A, 7A, 19A, 9B, 15B
Homeopathic treatments & non-herbal systemic complementary medicine (vitamin C, melatonin,…)**	21A, 25A, 42A, 6B, 7B, 13B, 14B, 21B, 22B, 23B, 24B, 25B, 31B
General articles on interactions in oncology	10A, 24A

* see Supplementary Data S3 for references. As and Bs refer to part A and part B of those references

**No pharmacokinetic interaction was detected with these substances
